# Ein Vergleich von 4 konvolutionalen neuronalen Netzen in der histopathologischen Diagnostik von Speicheldrüsenkarzinomen

**DOI:** 10.1007/s00106-023-01276-z

**Published:** 2023-02-03

**Authors:** Tobias Schulz, Christoph Becker, Gian Kayser

**Affiliations:** 1 Klinik für Hals-Nasen-Ohrenheilkunde, Kopf- und Halschirurgie, Medizinische Fakultät, Albert-Ludwigs-Universität Freiburg, Killianstr. 5, 79106 Freiburg, Deutschland; 2 Gemeinschaftspraxis für Pathologie, Freiburg, Deutschland

**Keywords:** Maschinelles Lernen, Algorithmen, Künstliche Intelligenz, Automatisierte Mustererkennung, Klassifikation, Machine learning, Algorithms, Artificial intelligence, Automated pattern recognition, Classification

## Abstract

**Hintergrund:**

Maligne Speicheldrüsentumoren sind aufgrund ihrer großen Anzahl an histopathologischen Entitäten, ihres seltenen Auftretens und der Vielfalt der klinischen und histologischen Präsentation eine besondere Herausforderung in der Diagnostik. Ziel der vorliegenden Arbeit ist es, die Anwendung von konvolutionalen neuronalen Netzen (CNN) als Hilfsmittel bei der histologischen Diagnose von malignen Speicheldrüsentumoren zu untersuchen und zu vergleichen.

**Methoden:**

Dazu wurden 118 histologische Schnitte von Speicheldrüsenkarzinompräparaten von 68 Patienten hochauflösend digitalisiert. Diese virtuellen Schnitte wurden in kleine Bildausschnitte unterteilt. Die 83.819 Bilder wurden in 4 Kategorien eingeteilt: Hintergrund, Binde- und Stützgewebe, nichtneoplastisches Speicheldrüsengewebe und Speicheldrüsenkarzinomgewebe, wobei unter der letzten Kategorie die Entitäten adenoidzystisches Karzinom, Adenokarzinom („not otherwise specified“), Azinuszellkarzinom, Basalzellkarzinom, Mukoepidermoidkarzinom und das myoepitheliale Karzinom zusammengefasst wurden. Die kategorisierten Bilder wurden dann in einem Trainings‑, Validierungs- und Testlauf von mit dem Bilddatensatz ImageNet vortrainierten CNN (Inception ResNet v2, Inception v3, ResNet152, Xception) in verschiedenen Pixelgrößen verarbeitet.

**Ergebnisse:**

Die Accuracy-Werte reichten von 18,8–84,7 % über alle Netzarchitekturen und Pixelgrößen, dabei erreichte das Inception-v3-Netz den höchsten Wert bei 500 × 500 Pixel. Die erreichten Recall-Werte bzw. die Sensitivität für verschiedene Pixelgrößen lagen bei bis zu 85 % (Inception-v3-Netz bei 1000 × 1000 Pixel). Der minimal erreichte F1-Score misst 0,07 für das Inception ResNet v2 sowie das Inception v3 bei jeweils 100 × 100 Pixeln, der maximal erreichte F1-Score lag bei 0,72 für das Xception bei 1000 × 1000 Pixeln. Das Netz mit den kürzesten Trainingszeiten war das Inception v3, das allen anderen getesteten Netzen bei jeder Pixelgröße überlegen war.

**Schlussfolgerung:**

In der vorliegenden Arbeit konnte erstmals für den Bereich der histopathologischen Analyse von Speicheldrüsenhistologien die Anwendbarkeit von CNN dargestellt und ein Vergleich der Performance verschiedener Netzarchitekturen angestellt werden. Die Ergebnisse lassen einen deutlichen potenziellen Nutzen zukünftiger Anwendungen erkennen.

## Abstract (en)

**Background:**

Malignant salivary gland tumors represent a particular diagnostic challenge due to the large number of histopathological entities, their rare occurrence, and the diverse clinical and histological presentations. The aim of this work is to investigate and compare convolutional neural networks (CNNs) as a diagnostic tool for histological diagnosis of salivary gland cancer.

**Methods:**

From salivary gland cancer preparations of 68 patients, 118 histological slides were digitized at high resolution. These virtual sections were then divided into small image sections, and the resultant 83,819 images were sorted into four categories: background, connective tissue, non-neoplastic salivary gland tissue, and salivary gland cancer tissue. The latter category grouped the entities adenoid cystic carcinoma, adenocarcinoma (not otherwise specified), acinar cell carcinoma, basal cell carcinoma, mucoepidermoid carcinoma, and myoepithelial carcinoma. The categorized images were then processed in a training, validation, and test run by the ImageNet pretrained CNN frameworks (Inception ResNet v2, Inception v3, ResNet152, Xception) in different pixel sizes.

**Results:**

Accuracy values ranged from 18.8% to 84.7% across all network architectures and pixel sizes, with the Inception v3 network achieving the highest value at 500 × 500 pixels. The recall values/sensitivity reached up to 85% for different pixel sizes (Inception v3 network at 1000 × 1000 pixels). The minimum F1 score achieved was 0.07 for the Inception ResNet v2 and the Inception v3 at 100 × 100 pixels each, the maximum F1 score achieved was 0.72 for the Xception at 1000 × 1000 pixels. Inception v3 was the network with the shortest training times, and was superior to all other networks at any pixel size.

**Conclusion:**

The current work was able to demonstrate the applicability of CNNs for histopathological analysis of salivary gland tumors for the first time and provide a comparison of the performance of different network architectures. The results indicate a clear potential benefit for future applications.

## Computerbasierter Lernprozess

Die Analyse mittels neuronaler Netze stellt einen computerbasierten Lernprozess dar, bei dem anhand von Beispielen gelernt wird. Im Anschluss wird die erlernte Erfahrung auf unbekannte Daten verallgemeinert. Eine Architektur dieser künstlichen neuronalen Netze ist das gefaltete neuronale Netz, das „convolutional neural network“ (CNN), das sich allem voran bei der Analyse von Bild- und Sprachdaten als besonders effektiv gezeigt hat und dort vorwiegend zur Anwendung kommt [1–3].

Im Verarbeitungsprozess durch die CNN findet zwischen dem Input und Output der Daten eine „convolution operation“ statt, die grundlegend die Leistungsfähigkeit eines CNN bestimmt. Darunter versteht man die Verarbeitung der Daten über mehrere Ebenen durch eine Art Filter, die der Mustererkennung dient. Das reicht von einfachen geometrischen Figuren wie Linien oder Kreisen bis hin zu histologischen Merkmalen wie Zellkernen oder komplexeren Merkmalen wie Augen. Während des Lernprozesses passen sich diese Ebenen („layer“) durch einen sog. Feed-forward-Prozess, durch den die Gewichtung der einzelnen Neuronen aufgrund komplexer statistischer Berechnungen auf die Zielgruppierung hin angepasst wird, immer genauer an den Trainingsdatensatz an, um in einem Validierungslauf dieses Erlernte auf neue Daten anzuwenden. Grundsätzlich unterscheiden sich die verschiedenen CNN durch die Anzahl und Architektur der genannten Ebenen und deren Verbindungen (d. h. „convolutional layer“, „pooling layer“, „fully-connected layer“) [4, 5].

Ein großes Feld stellen dabei die Möglichkeiten in der Klassifikation und Tumordetektion bei histologischen Bildern im Bereich der digitalen Pathologie dar. Vorangehende Arbeiten konnten bereits für verschiedene Organsysteme die Möglichkeit einer zuverlässigen Klassifikation histologischer Merkmale bzw. Tumorentitäten darstellen, so z. B. beim Mammakarzinom [6, 7] und kolorektalen Karzinom [8, 9]. Auch im Bereich der histologischen Klassifikation von Speicheldrüsentumoren und Karzinomen im Kopf-Hals-Bereich sind in jüngster Zeit Publikationen erschienen, die die Anwendbarkeit von CNN auch in diesem Feld suggerieren [10–12].

Maligne Speicheldrüsentumoren sind aufgrund ihrer Vielzahl an histopathologischen Entitäten und ihres seltenen Auftretens eine besondere Herausforderung in der feingeweblichen Diagnostik. Nach der vierten Auflage der Klassifikation der Weltgesundheitsorganisation (WHO) zu Speicheldrüsentumoren aus dem Jahr 2017 gibt es allein 20 verschiedene histologische Karzinomentitäten [13].

In der vorliegenden Arbeit haben die Autoren daher die Leistungsfähigkeit unterschiedlicher CNN im Bereich der Tumordetektion und histologischen Klassifikation von Speicheldrüsenkarzinomen untersucht und miteinander verglichen. Die Etablierung eines entsprechenden zuverlässigen Algorithmus in diesem Bereich wäre mit einer Arbeitsentlastung, Zeitersparnis bei der Diagnosefindung und letztlich einer schnelleren und besseren Behandlung von Patientinnen und Patienten mit Speicheldrüsenkarzinomen verbunden.

## Methoden

### Datensatz

Es wurden 118 histologische Schnitte von Tumorpräparaten von 68 Patienten verwendet, die im Zeitraum vom 01.01.2003 bis zum 31.12.2018 im Universitätsklinikum Freiburg in der Klinik der Hals‑, Nasen- und Ohrenheilkunde operiert und anschließend im Institut für Klinische Pathologie aufbereitet und diagnostiziert wurden. Pro Patient wurden ein bis zwei Hämatoxilin-Eosin-gefärbte histologische Schnittpräparate hochauflösend digitalisiert (Panoramic Scan 150, Fa. 3DHistech, Budapest, Ungarn).

### Verarbeitungsprozess

Die digitalisierten histologischen Schnitte (virtuelle Schnitte, VS) wurden dann durch das Bildbearbeitungsprogramm QuPath [14] komplett durch gleichmäßige Rasterung in Einzelbilder unterteilt. Aus einem Schnitt gingen ungefähr 3500–7500 Einzelbilder hervor, insgesamt entstanden knapp 550.000 Einzelbilder mit einer Größe von 1000 × 1000 Pixel (entsprechend 230 × 230 µm).

**Figure 1:**
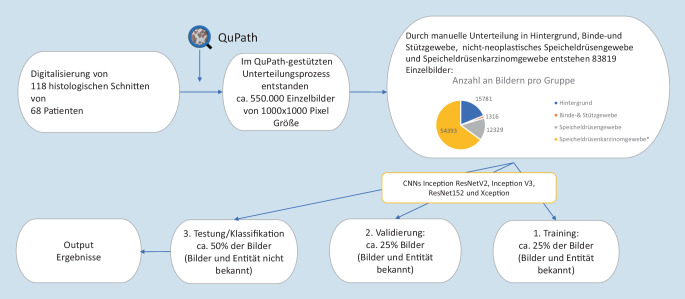

Für diesen Versuchsaufbau wurden die knapp 550.000 Bilder erneut in die auf dem Bild vorkommenden 4 Gewebetypen sortiert: Hintergrund, Binde- und Stützgewebe, nichtneoplastisches Speicheldrüsengewebe und Speicheldrüsenkarzinomgewebe, wobei unter der letzten Kategorie die Entitäten adenoidzystisches Karzinom, Adenokarzinom („not otherwise specified“), Azinuszellkarzinom, Basalzellkarzinom, Mukoepidermoidkarzinom und das myoepitheliale Karzinom zusammengefasst wurden. Diese Unterteilung erfolgte manuell durch einen geschulten Arzt unter Supervision und Überprüfung durch einen langjährig tätigen Facharzt für Pathologie. Auf dem Bild mussten ≥ 90 % desjenigen Gewebetyps zu sehen sein, um in die entsprechende Kategorie sortiert zu werden. Es durften keine Färbe- oder Digitalisierungsfehler zu sehen sein. So entstanden 83.819 Einzelbilder in hoher Qualität. Diese sortierten Bilder wurden dann in einem Trainings‑, Validierungs- und Testlauf von den etablierten und mit ImageNet vortrainierten CNN Inception ResNet v2, Inception v3, ResNet152 und Xception in 4 verschiedenen Pixelgrößen (100 × 100; 250 × 250; 500 × 500; 1000 × 1000) verarbeitet. Die einzelnen Pixelgrößen wurden via Downscaling vor Verarbeitung durch die CNN erstellt. Der Trainings- und Validierungslauf bestand dabei aus jeweils 2000 Bildern pro Gruppe. Die Berechnungen wurden auf einem Alienware R8 PC (Dell, Round Rock, Texas, USA), Intel Core i9 9900, 64 GB RAM (Santa Clara, Kalifornien, USA) mit einer NVIDIA-GeForce-2080Ti-Grafikkarte (Nvidia, Santa Clara, Kalifornien, USA) mit 11 GB RAM durchgeführt (Abb. 1).

### Bewertung der neuronalen Netze

Wie erfolgreich die CNN die gestellte Aufgabe bewältigen, bemisst sich maßgeblich nach etablierten Gütemaßen, die hier im Folgenden erläutert werden:

Im „machine learning“ ist die Accuracy definiert als der Anteil von korrekten Vorhersagen an allen getroffenen Vorhersagen. Der Recall-Wert entspricht dem Quotient aus richtig-positiven Testergebnissen und der Summe aus richtig-positiven und falsch-negativen Testergebnissen.

Der F1-Score fasst die Precision und den Recall-Wert eines Klassifikators in einer einzigen Metrik zusammen, indem er deren harmonisches Mittel bildet. Er wird in erster Linie verwendet, um die Leistung von 2 Klassifikatoren oder eben auch CNN zu vergleichen. Die „epoch times“ bezieht sich auf die Zeit, die die Netze für ihre Trainingszyklen durch den gesamten Trainingsdatensatz benötigten.

## Ergebnisse

**Figure 2:**
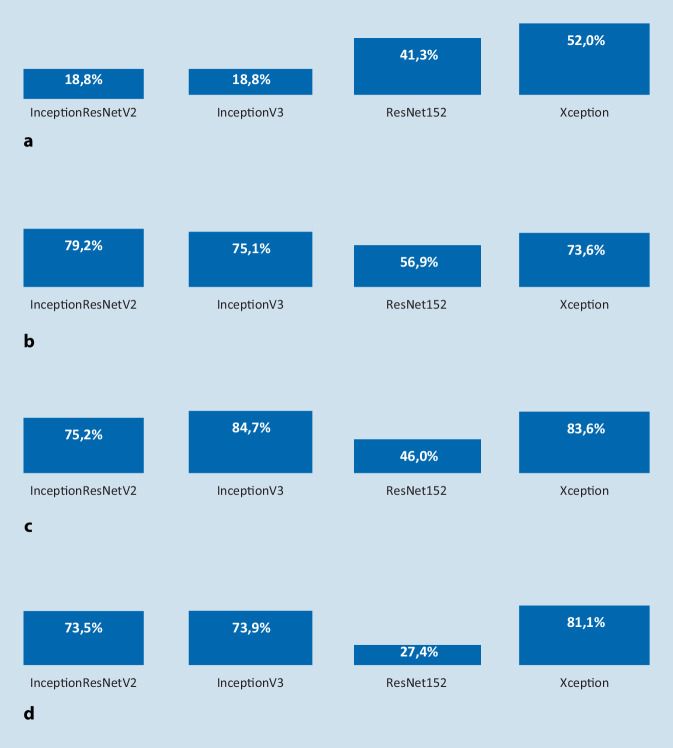

In der Abb. 2a–d sind die Accuracy-Werte der verwendeten CNN mit verschiedenen Pixelgrößen dargestellt.

Die Werte reichten im Vergleich zwischen den verschiedenen Netzarchitekturen von 84,7 % beim Inception v3 bei einer Pixelgröße von 500 bis hin zu 18,8 % beim Inception ResNet v2 und Inception v3 bei jeweils einer Pixelgröße von 100.

**Figure 3:**
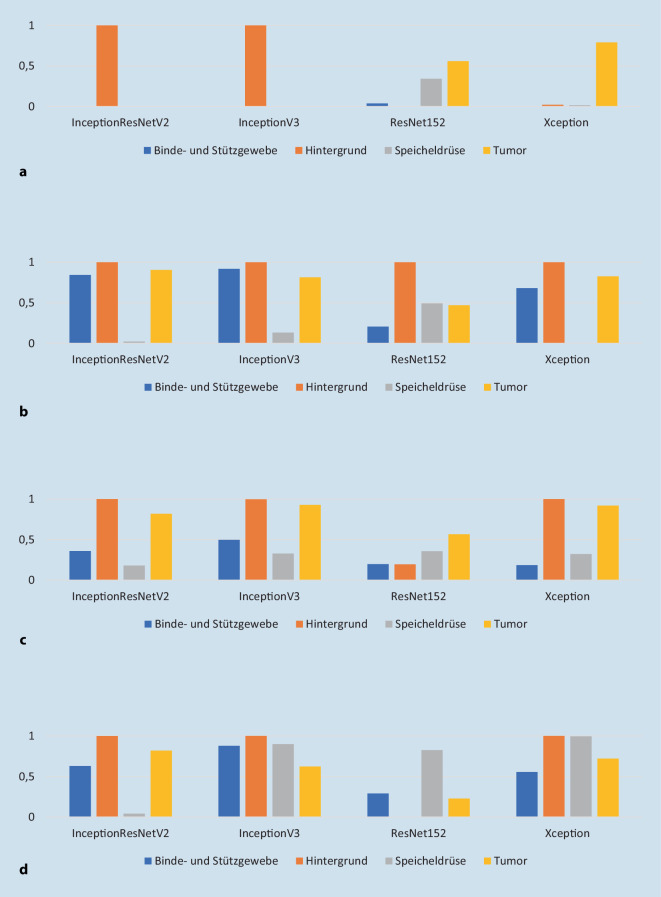

In Abb. 3a–d finden sich die Recall-Werte bzw. die Sensitivität für die verschiedenen CNN und Pixelgrößen. Die Werte liegen zwischen 20 und 85 %.

Der minimal erreichte F1-Score maß 0,07 für das Inception ResNet v2 sowie für das Inception v3 bei jeweils 100 × 100 Pixeln, der maximal erreichte F1-Score lag bei 0,72 für das Xception bei 1000 × 1000 Pixeln. Den besten durchschnittlichen Wert über alle Pixelgrößen hinweg erreichte das Xception-CNN mit einem Wert von 0,5.

In Tab. 1 ist die Trainingszeit der verschiedenen CNN in Stunden aufgeführt. Das Netz mit den kürzesten Trainingszeiten war das Inception v3, das allen anderen Netzen bei jeder Pixelgröße in der Berechnungszeit überlegen war. Das Netz mit den längsten Trainingszeiten ist das Inception ResNet v2, das im Vergleich zum Inception v3 bei einer Pixelgröße von 1000 × 1000 knapp 6‑mal so viel Zeit für den Trainingslauf benötigte.

**Table 1:** 

CNN	Pixelgröße	„Epoch times“ (h)^a^
Inception ResNet v2	100 × 100	0,23
	250 × 250	1,56
	500 × 500	7,19
	1000 × 100	43,26
Inception v3	100 × 100	0,07
	250 × 250	0,37
	500 × 500	1,72
	1000 × 100	7,41
ResNet152	100 × 100	0,35
	250 × 250	1,73
	500 × 500	7,08
	1000 × 100	33,35
Xception	100 × 100	0,11
	250 × 250	0,63
	500 × 500	2,59
	1000 × 100	11,33

^a^Aufgezeigt wird die zusammengefasste Zeit in Stunden, die die Netze für das Training benötigten, dabei entspricht ein „epoch“ einem Trainingszyklus. Es wurden jeweils 20 Trainingszyklen durchlaufen

## Diskussion

Die Auswahl der vier CNN erfolgte auf Basis der ImageNet Large Scale Visual Recognition Challenge (ILSVRC), eines etablierten Wettbewerbs, der regelmäßig die leistungsfähigsten Netze im Bereich der Bilderkennung gegeneinander antreten lässt, sowie aus vorangegangenen Arbeiten zu CNN im medizinischen Bereich. Da im Bereich der histopathologischen Speicheldrüsendiagnostik mittels CNN noch kaum Arbeiten publiziert sind, war der Versuch, verschiedene Netze zu vergleichen, um eine möglichst leistungsfähige Netzarchitektur für zukünftige Arbeiten herauszufinden, besonders relevant.

### Accuracy

**Figure 4:**
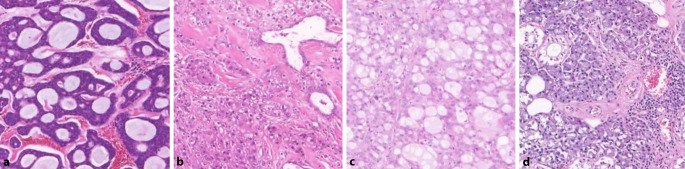

Die Accuracy erreichte Werte zwischen 18,8 und 84,7 %, was einen Aufschluss über die Komplexität der verarbeiteten Daten und die Herausforderung im Bereich der Diagnostik von Speicheldrüsengeweben gibt. So sind unter der Kategorie „Tumor“ den CNN 6 verschiedene Tumorentitäten als Bilddateien zur Analyse angeboten worden – mit sehr heterogener histologischer Darstellung (Abb. 4) einerseits, andererseits mit teils großer histologischer Ähnlichkeit zu normalem, nichtneoplastischem Speicheldrüsengewebe.

In vorangegangenen Arbeiten von Esteva et al. [15] und Jeyaraj et al. [16] konnten Accuracy-Werte von 72,1 bzw. 91,4 % erreicht werden, wobei jeweils die Architektur des Inception v3 verwendet wurde. Das Inception v3 zeigte auch in der hier vorliegenden Arbeit den besten Accuracy-Wert bei 500 × 500 Pixeln. In den genannten Arbeiten wurden Pixelgrößen von 299 × 299 bzw 250 × 250 verwendet. In der hier vorliegenden Arbeit konnte bei 250 × 250 Pixeln das Inception ResNet v2 mit einer Accuracy von 79,2 % den besten Wert erreichen.

Es lässt sich somit zeigen, dass sich bei verschiedenen Pixelgrößen auch deutliche Unterschiede in der Klassifikations-Accuracy ergeben. Es sollte somit in zukünftigen Arbeiten der Versuch unternommen werden, die Performance der jeweiligen CNN-Architekturen bei verschiedenen Pixelgrößen zu prüfen, um optimale Ergebnisse des Algorithmus zu erzielen.

### Vergleichskriterien beim Deep Learning

In den Arbeiten von Esteva et al. [15] und Jeyaraj et al. [16] konnten Recall-Werte von maximal 96 % bzw. 94 % erzielt werden. In der vorliegenden Arbeit lagen die Werte zwischen 20 (Xception 100 × 100 Pixel) und 85 % (Inception v3 1000 × 1000). Diese große Varianz der Werte lässt sich vor allem durch ein sog. Overfitting der Netzarchitektur während des Lernprozesses und die Komplexität der Aufgabe erklären. Dabei lernt das Netz beispielsweise ganz bestimmte Merkmale als Distinktionskriterium für einen Gewebetyp während des Lernprozesses. In der Testphase ist das Netz dann aber nicht fähig, die neuen Daten richtig einzuordnen, da die erlernten Distinktionskriterien keine hinreichend guten verallgemeinerbaren Merkmale darstellen, um noch unbekannte Bilder dieses Gewebetyps korrekt zu klassifizieren.

Insgesamt müssen beim Vergleich dieser Arbeiten Einschränkungen wie die Größe des Datensatzes (Esteva et al. 129.450 vs. 700 bei Jeyaraj et al. vs. 83.819 Bilder bei der hier vorliegenden Arbeit), die Komplexität der einzelnen Bilder und die genaue Aufgabe der Klassifikation bedacht werden. Bei der Arbeit von Jeyaraj et al. war die Aufgabe an den Algorithmus auf die Differenzierung zwischen malignen und benignen Befunden beschränkt, wohingegen bei der hier vorliegenden Arbeit 4 verschiedene Klassifikationsmöglichkeiten gefordert wurden. Dieser Umstand erhöht die Komplexität der Aufgabe um ein Vielfaches und führt entsprechend auch zu niedrigeren Recall-Werten. In der Arbeit von Esteva et al. dagegen sollte der Algorithmus zwischen 3 verschiedenen Möglichkeiten klassifizieren: malignen, benignen und nichtneoplastischen Befunden. Dafür waren im Trainingsset für die nichtneoplastischen Befunde allein 10 sehr verschiedene Entitäten als Datensatz gewählt worden. Im Vergleich dazu waren im Trainingsset in dem Versuchsaufbau der vorliegenden Arbeit für nichtneoplastisches Speicheldrüsengewebe für die Algorithmen auch nur Bilder von nichtneoplastischem Speicheldrüsengewebe und Binde- und Stützgewebe zu erlernen, was die Aufgabe vereinfacht. Diese Beispiele zeigen, dass der direkte Vergleich verschiedener Arbeiten im Spektrum des Deep Learning mit CNN im Detail sehr genau betrachtet werden muss.

Mit dieser Arbeit konnten die Autoren zeigen, dass auch im Bereich der histologischen Klassifikation von Speicheldrüsenkarzinomen mit CNN eine vergleichbar gute Leistungsfähigkeit erreicht werden kann. Das leistungsfähigste Netz in dieser Arbeit war das Xception-CNN, das die höchsten durchschnittlichen Accuracy-Werte mit 72,6 % und den besten durchschnittlichen F1-Score mit 0,5 über alle Pixelgrößen hinweg erreichte und damit die stabilste Performance zeigte. Die besten durchschnittlichen Accuracy-Werte mit 72,4 % und den besten durchschnittlichen F1-Score mit 0,54 über alle Netze hinweg konnte bei der Pixelgröße von 500 × 500 Pixeln erreicht werden, wo auch der beste einzelne Accuracy-Wert mit dem Inception-v3-CNN mit 84,7 % erreicht werden konnte. Es sollte daher in folgenden Arbeiten jeweils auch eine Pixelgröße von 500 × 500 Pixeln geprüft werden.

## Ausblick

In zukünftigen Arbeiten könnte die Klassifikationen in einzelne Tumorentitäten erfolgen. Um alle 20 Karzinomentitäten darzustellen, sind interinstitutionelle Kooperationen sinnvoll, um eine entsprechend große Datenkohorte aufzubauen und dadurch dem Ziel der computergestützten Diagnostikassistenz näher zu kommen und das komplette Potenzial ggf. auch für die Prognose- und Therapieprädiktion auszuschöpfen.

Die Ergebnisse der vorliegenden Arbeit lassen einen deutlichen potenziellen Nutzen zukünftiger Anwendungen erkennen; bis zur Translation in den klinischen Alltag braucht es jedoch noch weitere Studien und möglichst multizentrische Ansätze, um repräsentativ die Analyse aller Entitäten an Speicheldrüsenhistologien abbilden zu können.
